# High pump depletion second-harmonic generation using domain engineered thin-film lithium niobate waveguides

**DOI:** 10.1515/nanoph-2025-0505

**Published:** 2025-11-19

**Authors:** Chenyu Wang, Mengwen Chen, Xiao-Hui Tian, Zishuo Gu, Jie Tang, Yong Zhang, Zikang Wang, Kunpeng Jia, Chenyang Shi, Xiaowen Gu, Guang Qian, Zhenlin Wang, Shi-Ning Zhu, Zhenda Xie

**Affiliations:** National Laboratory of Solid State Microstructures, School of Electronic Science and Engineering, College of Engineering and Applied Sciences, School of Physics, and Collaborative Innovation Center of Advanced Microstructures, Nanjing University, Nanjing 210093, China; National Key Laboratory of Solid-State Microwave Devices and Circuits, Nanjing Electronic Devices Institute, Nanjing 210016, China

**Keywords:** TFLN, quasi-phase matching (QPM), frequency conversion, pump depletion

## Abstract

Thin-film lithium niobate (TFLN) has emerged as a powerful platform for integrated nonlinear optics owing to its large *χ* ^(2)^ nonlinearity, tight confinement and flexible tunability. To fully excavate such superior nonlinear optical properties, domain engineering is commonly adopted to fulfill the phase matching condition of *χ* ^(2)^ processes. During the past decade, various domain engineered TFLN nonlinear optical devices have been demonstrated, showing extremely high length-normalized nonlinear optical conversion efficiencies. However, application-driven scenarios demand absolute energy conversion in nonlinear frequency conversion rather than length-normalized efficiencies, but the progress has been limited by imperfect fabrication processes. In this work, we realize effective on-chip nonlinear energy conversion by developing low-loss and high-quality domain engineered TFLN waveguides with long interaction length. Ion beam trimming (IBT) technique and an etching-prior-poling workflow are adopted for such fabrication. Optical characterization yields an overall second-harmonic generation (SHG) efficiency of 2,590 %/W. A high pump depletion of 85.7 % is demonstrated under continuous-wave operation, which directly reflects strong nonlinear energy conversion. These results may lead to breakthroughs in applications like classical optical frequency conversion, quantum frequency conversion, and quantum light generation.

## 1 Introduction

Photonic integrated circuits (PICs) are under sustained pressure to deliver higher efficiency, lower power, tighter footprints and foundry-ready solutions [1], [2]. Among emerging PIC platforms, TFLN stands out for its strong *χ* ^(2)^ nonlinearity (*d*_33_ = 27 pm/V), tight modal field confinement, low optical loss, flexible dispersion engineering and wafer-scale fabrication ability [3], [4], [5], [6]. On TFLN, *χ* ^(2)^-based devices have demonstrated excellent performance in SHG [7], [8], [9], [10], difference frequency generation (DFG) [11], [12], sum frequency generation (SFG) [13], [14], [15], [16], spontaneous parametric down conversion (SPDC) [17], [18], [19], optical parametric amplification (OPA) [20], [21], [22] and optical parametric oscillation (OPO) [23], [24], [25], [26] – covering applications from classical wavelength conversion to quantum light sources [27], [28], [29]. Compared with *χ* ^(3)^-based (Kerr effect, spontaneous four-wave mixing, etc.) devices, *χ* ^(2)^ interactions are typically orders of magnitude stronger [30], enabling lower pump thresholds and higher interaction efficiencies, while also supporting broader frequency spans from ultraviolet to radio frequency [31].

To fully harness these advantages, efficient phase matching is indispensable, including birefringent phase matching [32], modal phase matching [33], [34], [35], cavity phase matching [36], [37] and quasi-phase matching (QPM) [38], [39], wherein QPM is usually the first choice on TFLN. By periodically modulating the nonlinear coefficient along the propagation direction, QPM enables interactions among fundamental modes with maximal spatial overlap and exploitation of its largest tensor element *d*_33_ on TFLN [40], [41]. This modulation is typically realized by periodic ferroelectric domain inversion (periodic poling) using lithographically defined electrodes.

While QPM on TFLN has delivered impressive figures of merit, most prior reports emphasize length-normalized efficiencies [15], [42] or low-power overall efficiencies [39] as primary small-signal metrics for SHG and related processes. However, many application-driven scenarios ultimately hinge on absolute nonlinear energy conversion and low noise, such as cascaded *χ* ^(2)^ processes [43], mid-IR DFG sources [11], [24], and single-photon quantum frequency conversion [13], [44]. Reported absolute conversion in nanophotonic TFLN waveguides with QPM was typically 9–62 % [9], [15], [45], [46], [47], [48], [49]. In practice, achieving strong on-chip nonlinear energy conversion remains nontrivial due to propagation loss, nonuniformity in poling quality and sensitivity of the phase matching condition to fabrication-induced geometry variations – even when the length-normalized/overall efficiency is high in the metrics of undepleted pump. In recent years, an adapted poling strategy using electron-beam lithography has been shown to overcome nanoscale inhomogeneity and achieve 82.5 % single-pass absolute conversion efficiency in nanophotonic TFLN waveguides [39].

In this paper, we pursue achieving absolute nonlinear energy conversion in a domain engineered TFLN rib waveguide using a deep-ultraviolet (DUV) compatible fabrication. We develop low-loss and high-quality domain engineered TFLN waveguides with long interaction lengths by adopting IBT technique and an etching-prior-poling workflow [20]. High-fidelity domain patterns with duty cycles close to 50 % and low propagation loss on the order of 0.1 dB/cm are achieved simultaneously. Optical characterization yields an overall SHG efficiency of 2,590 %/W. A high pump depletion of 85.7 % is demonstrated under continuous-wave operation, which directly reflects strong nonlinear energy conversion. These results can provide a performance baseline for classical and quantum frequency conversion applications.

## 2 Theory and design

Nonlinear frequency conversion is governed by energy conservation and phase matching – the requirement that the generated fields add coherently along the waveguide. In general, any dispersive platform suffers from phase mismatch since material and modal dispersion lead to unequal phase velocities, thereby suppressing net energy transfer. QPM is a general momentum-engineering method in which the nonlinear coupling is spatially modulated to supply a reciprocal superlattice vector that compensates the intrinsic phase mismatch. In TFLN, the uniaxial spontaneous polarization **P**_s_ can point either along +*z* or −*z*, giving rise to two ferroelectric states separated by domain walls (DWs). By periodically inverting these domains in a waveguide, one forms a one-dimensional superlattice with period Λ, whose reciprocal superlattice vector is expressed as (magnitude, scalar)

(1) $$G_{m}=\frac{2\pi m}{\Lambda }\;\left(m\in Z\right)$$

This superlattice vector supplies the momentum needed to compensate the intrinsic phase mismatch and restores coherent growth of nonlinear polarization.

**Figure 1:**
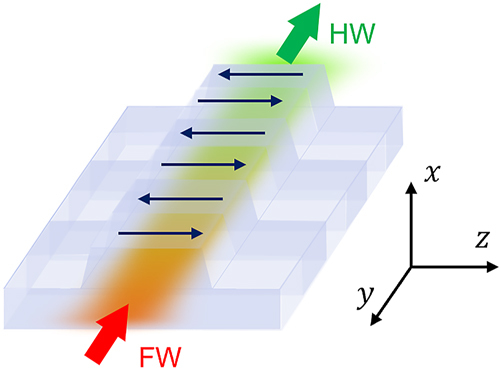
Schematic illustration of frequency conversion in an *x*-cut periodically poled TFLN waveguide. The dark blue arrows represent **P**_s_ orientations. FWs can be effectively converted to harmonic waves (HWs) under QPM.

Referring to Figure 1, we focus on SHG. With energy conservation *ω*_SH_ = 2*ω*_FW_, where the subscript FW denotes the fundamental wave and SH denotes the second harmonic, the waveguide phase mismatch is Δ*k* ≡ *k*_SH_ − 2*k*_FW_. QPM enforces an effective condition

(2) $$\Delta k_{\text{eff}}=\Delta k-G_{m}=0$$

To realize efficient QPM in TFLN, we must use FW and SH modes both polarized along the optical *z*-axis so as to access the maximum tensor component *d*_33_, and ensure that both are the fundamental transverse modes for maximal spatial overlap. Under the periodic modulation of *d*_33_ via periodic domain inversion, the effective nonlinear coefficient is given by

(3) $$d_{\text{eff}}^{\left(m\right)}=\frac{2}{m\pi }\;d_{33}\;\sin\left(m\pi D\right)$$

where the duty cycle *D* denotes the fraction of one period Λ occupied by domains polarized along +*z*. The design principle then is to choose *m* = 1 and *D* = 0.5 to maximize $\left|d_{\text{eff}}\right|$ , while processing must minimize propagation loss. These criteria guide our layout, fabrication and characterization strategy in the following sections.

On TFLN, periodic poling with lithographically patterned electrodes, i.e., batch ferroelectric domain inversion driven by high *E*-fields, has become the mainstream QPM implementation. Although theoretical models predict high conversion efficiency, the practical performance is often limited by fabrication imperfections, including propagation loss, imperfection in the poling duty cycle or quality, and the sensitivity of phase-matching condition to small geometry deviations (etch depth, waveguide width, film thickness) along the interaction length. Specifically, film thickness variation is the primary contributor to perturb Δ*k*, which degrades conversion efficiency [50]. To mitigate this issue and achieve effective energy conversion in nonlinear frequency conversion, adaptive poling [39] and IBT [20], [51], [52] techniques were proposed and demonstrated. IBT employs a scanned and focused ion beam with dwell time control to remove material locally at the nanometer level across the wafer [53], thereby suppressing thin-film thickness variation. In this work, we choose IBT because this technique enables compatibility with our wafer-scale fabrication.

## 3 Experimental results

### 3.1 Fabrication

**Figure 2:**
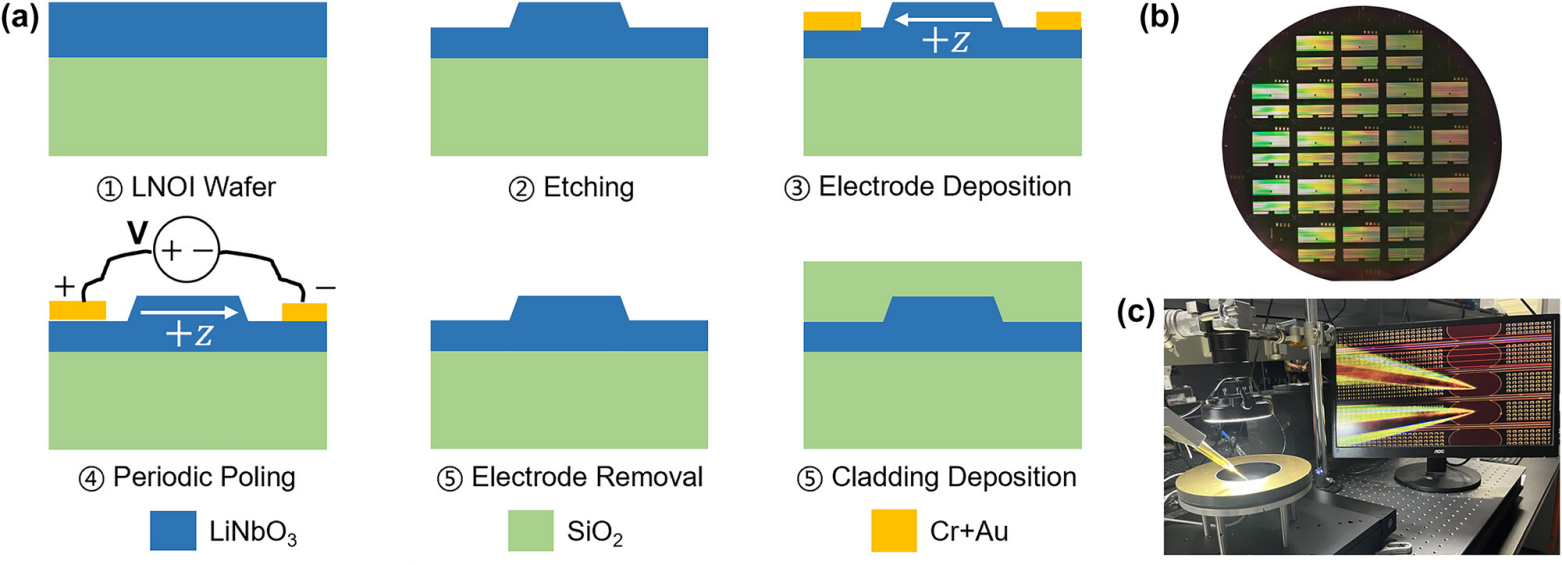
Waveguide fabrication with etching-prior-poling on the *x*-cut TFLN wafer. (a) Schematic diagrams of *x*-cut periodically poled TFLN rib waveguide fabrication. The film thickness is 600 nm with waveguide etch depth 300 nm. (b) A photo of the 4-inch wafer after electrode deposition (step 3). (c) Automated programmable poling setup in step 4.

High-quality QPM in TFLN waveguides hinges on two fabrication operations: defining the waveguide geometry through etching and engineering ferroelectric domain inversion (poling). Historically, most devices have relied on poling-prior-etching because of its technical simplicity. However, the subsequent chemical etch can imprint periodic sidewall steps at antiparallel domains, increasing scattering loss hence degrading device performance. These shortcomings motivate the exploration of etching-prior-poling approaches [54], [55], [56], [57], [58], [59] which eliminate the root cause of selective-etch sidewall roughening through preserving the low-loss surface. We implement the workflow of etching-prior-poling on a 4-inch *x*-cut TFLN wafer shown in Figure 2(a). Rib waveguides are patterned using DUV lithography, exhibiting good overall etch uniformity across the wafer. The 600 nm film is dry-etched to 300 nm at an angle ∼67°, leaving a 300 nm slab. The periodic electrodes, composed of a 40 nm Cr adhesion layer topped by 30 nm of Au, are deposited by electron-beam evaporation and patterned by a lift-off process. Figure 2(b) shows the fabricated TFLN wafer. The positive and negative electrode tips are separated 6 µm across the waveguide without contacting the sidewalls to avoid triggering discharge-induced surface damage and the resulting excess loss. Electrode widths are adjusted according to QPM periods, and the corresponding duty cycles are controlled from 1/5 to 1/4. Periodic poling is then executed by applying high-voltage pulse trains across the electrode pairs to invert domains along the *z*-axis. Extending beyond single-device demonstrations, a computer-controlled poling station (Figure 2(c)) delivers programmable pulses, supporting rapid electrode pad addressing and automatic high voltage application across the whole wafer. Wafer-scale etching-prior-poling yields high domain-pattern fidelity. Such qualities are essential for meeting foundry-integration standards of high yield and reproducibility. The transmission loss is uniform across the wafer, on the order of 0.1 dB/cm.

**Figure 3:**
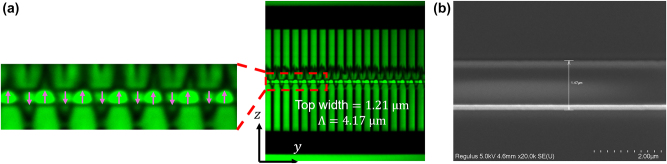
Microscopy of a periodically poled TFLN rib waveguide. (a) Confocal SH microscope image of a periodically poled rib waveguide (top width = 1.21 µm, Λ = 4.17 µm). The right panel shows the full electrode and waveguide region in the *z*-axis, while the left inset gives a magnified view of several periods, highlighting the inverted domains. The pink arrows represent the **P**_s_ orientation in each domain. (b) A scanning electron microscope (SEM) image of the periodically poled TFLN rib waveguide whose base width = 1.47 µm after the electrodes are removed.

Visualization of ferroelectric domains and examination of their quality are crucial for assessing whether QPM structures can deliver the intended optical performance. Nonlinear optical imaging techniques are particularly powerful in this context, since they directly probe the modulation of the *χ* ^(2)^ response. Confocal SH microscopy provides a noninvasive probe of ferroelectric domain patterns in TFLN. *χ* ^(2)^ flips signs between antiparallel domains, and the SH signal contrast is dominated by *d*_33_ [60]. Figure 3(a) displays a representative image of a periodically poled rib waveguide. The bright central band is the strip location of 600 nm thick LiNbO_3_, while the dimmer bands on the above and below correspond to the 300 nm thick slab. The pure black periodic comb-shaped lines and plates visible on either side of the bright and dim bands represent the metal electrodes. These electrodes are opaque to the confocal SH signal and thus appear dark. The inset further magnifies several poling periods, clearly revealing uniform contrast across successive domains with duty cycles approaching 50 %. The metal is removed after poling. An SEM image (Figure 3(b)) of a periodically poled TFLN rib waveguide taken after electrode cleansing shows smooth, continuous sidewalls and a clean slab without periodic terraces or corrugations. This morphology is consistent with our non-contact electrode geometry in our etching-prior-poling process, which indicates that the poling stage does not degrade the optical waveguide and aligns with the low-scattering performance evidenced in the subsequent measurements. Finally, a 2 µm thick SiO_2_ cladding is deposited on top of the wafer via plasma-enhanced chemical vapor deposition. We then cleave the wafer into individual chips, polish the end facets to optical quality, and perform optical performance tests on the polished waveguides.

### 3.2 Optical characterization

**Figure 4:**
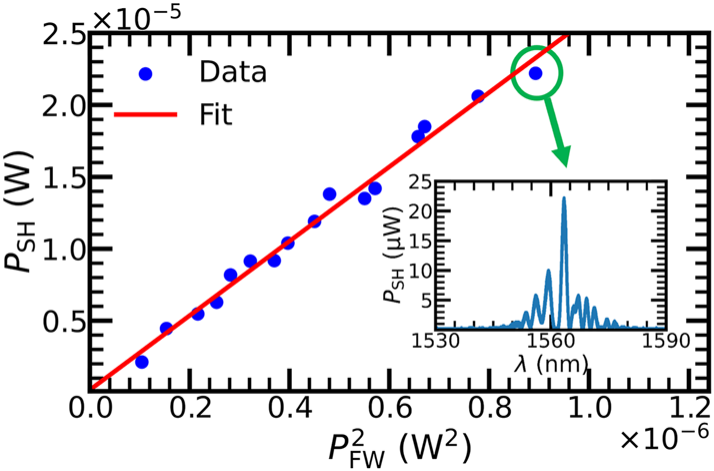
Overall SHG efficiency measurement with a linear fit in the low pump power regime. The inset shows the SH spectrum when the on-chip input FW power *P*_FW_ = 945 µW.

We have confirmed high-quality QPM domains in the waveguides via confocal SH microscopy. However, practical utility ultimately hinges on device-level performance. We measure SHG to assess the nonlinear conversion ability of a straight waveguide with a total length of 15 mm, of which 12.3 mm is periodically poled. The chip temperature was controlled near *T* = 79.8 °C throughout the measurement. In the low pump power regime where the undepleted pump assumption holds, the overall efficiency of an SHG process is defined as

(4) $$\eta =\frac{P_{\text{SH}}}{P_{\text{FW}}^{2}}\times 100\;\%$$

where *P*_FW_ and *P*_SH_ denote the FW and SH powers, respectively. The experimental result is shown in Figure 4. The SH power is recorded at different FW input levels using calibrated power meters, and a linear fit yields *η* = (2.59 ± 0.08) × 10^3^ % W^−1^. The inset shows the SH spectrum when *P*_F_ = 945 µW, with a principal peak reaching 22.2 μW at *λ*_FW_ = 1,563.447 nm. The current measured efficiency is mainly limited by unwanted asymmetric side peaks that may be attributed to film thickness variations along the waveguide or the deviation from a perfect 50 % duty cycle. This can also be improved via better fabrication in the future.

**Figure 5:**
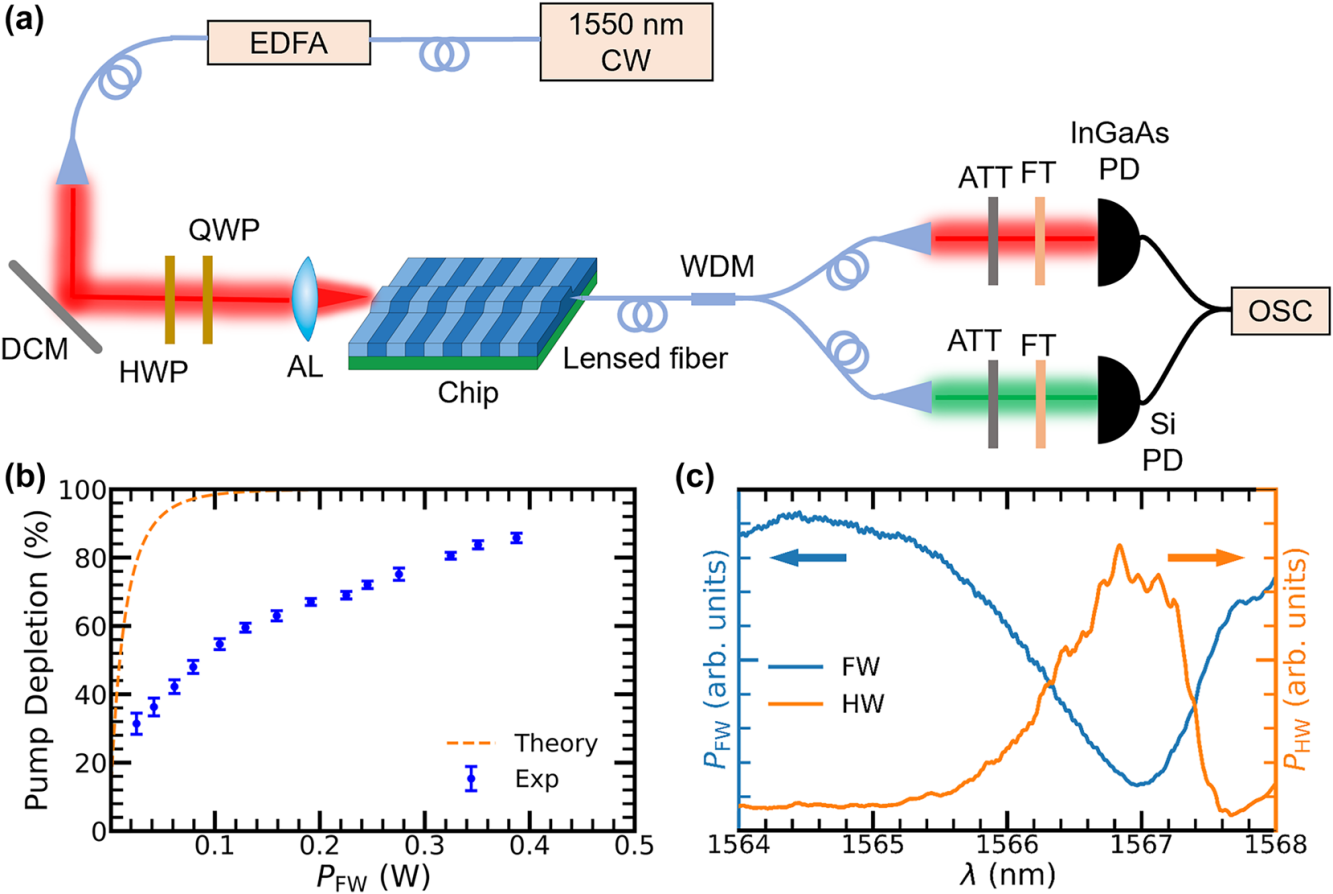
Experimental setup and results for pump depletion in SHG. (a) Experimental setup for high pump power SHG measurements. CW: continuous wave; EDFA: erbium-doped fiber amplifier; DCM: dielectric-coated mirror; HWP/QWP: half/quarter-wave plate; AL: aspheric lens; WDM: wavelength-division multiplexer/demultiplexer; ATT: attenuator; FT: filter; PD: photodetector; OSC: oscilloscope. (b) Pump depletion measurement in the high pump power regime, where the depletion ratio increases with on-chip input FW power *P*_FW_. Experimental data with error bars (Exp) and a theoretical limit (Theory) are plotted. (c) Spectral anticorrelation between FW dips and HW peaks when *P*_FW_ = 0.387 W.

To verify strong on-chip pump depletion beyond small-signal metrics, we conducted high-pump measurements on our straight waveguide device. In the high pump power regime, the undepleted pump approximation breaks down. According to energy conservation, the on-chip HW power and efficiency can be estimated through the FW power depletion ratio. The experimental setup for this measurement is illustrated in Figure 5(a). A continuous-wave (CW) 1,550 nm laser is amplified by an erbium-doped fiber amplifier (EDFA) with an isolator and coupled into the chip. A half-wave plate (HWP) and quarter-wave plate (QWP) are inserted before coupling to adjust the polarization, ensuring that the injected FW corresponds to the TE mode that participates in the nonlinear conversion. At the output facet, the converted beam is collected by a lensed fiber and sent to a wavelength-division multiplexer/demultiplexer (WDM), which separates the signal into two detection arms. In one arm, an InGaAs photodetector monitors the residual FW after passing through a long-pass filter that blocks wavelengths below 1,400 nm. In the other arm, a Si photodetector measures the generated SH after a short-pass filter that transmits only wavelengths below 1,000 nm. Both detection channels are equipped with attenuators (ATT) and filters (FT) to control optical power and suppress spurious background. During rapid scanning of the FW wavelength, the two photodetector (PD) signals are synchronously captured by an oscilloscope (OSC), enabling a direct comparison of FW depletion and SH growth under varying QPM conditions.

As the pump power increases, the pump depletion ratio can be observed as shown in Figure 5(b). Pump depletion keeps increasing within our maximal on-chip *P*_FW_ = 0.387 W, reaching a maximum value of (85.7 ± 1.4)%. No significant back-conversion has been observed when *P*_FW_ < 400 mW. The measured depletion remains below the ideal theoretical limit due to residual Δ*k* from wafer-scale thickness and fabrication nonuniformity and small deviations of the poling duty cycles from 50 %. Figure 5(c) shows the spectral correspondence between HW and FW. As the HW power exhibits its peaks, the FW transmission correspondingly dips – clear evidence of pump depletion at wavelengths where the SHG is phase-matched. This spectral anticorrelation between HW peaks and FW dips directly reflects efficient frequency conversion at those wavelengths.

## 4 Conclusions

In conclusion, we fabricate straight rib waveguides on a 4-inch *x*-cut TFLN wafer using DUV lithography, starting with a 600 nm film and etching 300 nm. The workflow combines this geometry with an etching-prior-poling process, which avoids sidewall roughness from selective etching and yields smooth rib and slab surfaces. Confocal SH microscopy confirms high-quality domain inversion with duty cycles close to 50 %, which preserves phase-matching efficiency and enables reproducible wafer-scale device performance. Optical tests on a 1.21 µm top width straight waveguide show an overall SHG efficiency as high as 2,590 %/W with propagation loss on the order of 0.1 dB/cm. Beyond the small-signal benchmark, under CW pumping the same device reaches 85.7 % pump depletion, directly evidencing strong on-chip nonlinear energy conversion without the aid of resonance enhancement. This achievement establishes pump depletion as a device-level performance metric for nonlinear energy conversion that complements conventional length-normalized efficiencies. Looking forward, further refinement of domain geometry and compensation of thickness-induced phase mismatch variations may push efficiencies even higher. Our methodology provides a scalable and foundry-compatible path for implementing *χ* ^(2)^-based frequency converters in both classical and quantum photonic systems.
